# The role of the cell wall in plant immunity

**DOI:** 10.3389/fpls.2014.00178

**Published:** 2014-05-06

**Authors:** Frederikke G. Malinovsky, Jonatan U. Fangel, William G. T. Willats

**Affiliations:** ^1^DNRF Center DynaMo and Copenhagen Plant Science Center, Department of Plant and Environmental Sciences, Faculty of Science, University of CopenhagenCopenhagen, Denmark; ^2^Department of Plant and Environmental Sciences, Faculty of Science, University of CopenhagenCopenhagen, Denmark

**Keywords:** plant cell wall, defense, PTI, PAMP, DAMP, callose, chitin, immunity

## Abstract

The battle between plants and microbes is evolutionarily ancient, highly complex, and often co-dependent. A primary challenge for microbes is to breach the physical barrier of host cell walls whilst avoiding detection by the plant’s immune receptors. While some receptors sense conserved microbial features, others monitor physical changes caused by an infection attempt. Detection of microbes leads to activation of appropriate defense responses that then challenge the attack. Plant cell walls are formidable and dynamic barriers. They are constructed primarily of complex carbohydrates joined by numerous distinct connection types, and are subject to extensive post-synthetic modification to suit prevailing local requirements. Multiple changes can be triggered in cell walls in response to microbial attack. Some of these are well described, but many remain obscure. The study of the myriad of subtle processes underlying cell wall modification poses special challenges for plant glycobiology. In this review we describe the major molecular and cellular mechanisms that underlie the roles of cell walls in plant defense against pathogen attack. In so doing, we also highlight some of the challenges inherent in studying these interactions, and briefly describe the analytical potential of molecular probes used in conjunction with carbohydrate microarray technology.

## INTRODUCTION

The dynamic interplay between pathogen and plant host is the product of millions of years of co-evolution. This struggle is often described in terms of an “arms race,” a fitting term considering the investment required and the significance for both sides. The goal for the plant is to keep healthy and fertile, but for pathogens the strategy varies. Biotrophic and hemibiotrophic microorganisms rely on keeping their host alive and unaware of their presence, at least until later stages of infection. In contrast, necrotrophs live and feed on dead and dying cells and can therefore use a more forceful attack strategy (Davidsson et al., 2013). The frontline of the plant defense system consists of physical and chemical barriers such as the cell wall, waxes, hairs, antimicrobial enzymes, and secondary metabolites. If these obstacles are overcome, the pathogen is still confronted by elaborate surveillance systems in which molecular sentinels operate to activate resistance responses (Jones and Dangl, 2006).

Molecular components that serve essential functions for the fitness or survival of microbes are often highly conserved. For plants, detection of such microbial fingerprints also referred to as pathogen-associated molecular patterns (PAMPs), is a warning of impending attack (Medzhitov and Janeway, 1997). Consequently, a key aspect of plant innate immunity is the ability to recognize PAMPs such as bacterial flagellin, lipopolysaccharides, peptidoglycans, and fungal chitin (Boller and Felix, 2009). In addition to sensing PAMPs, the ability to sense a compromised “self” by detecting damage-associated molecular patterns (DAMPs) such as released plant cell wall fragments is a central part of plant defense (Boller and Felix, 2009). Both PAMP- and DAMP-recognition activates PAMP-triggered immunity (PTI) which in general prevents microbial colonization. To escape detection and PTI induction, a common strategy among adapted microorganisms is to secrete a range of effector proteins that can modulate PTI components (Jones and Dangl, 2006). The stealth afforded by the microbial effectors can in turn be counteracted in the plant by an intracellular surveillance system consisting of an array of nucleotide-binding leucine-rich-repeat proteins that seek to detect the presence of such effector proteins, and enable induction of effector-triggered immunity (ETI). ETI is often associated with a localized cell death termed the hypersensitive response which functions to restrict spread of this more progressive stage of microbial attack (Jones and Dangl, 2006; Dodds and Rathjen, 2010). Hence the important feature of PTI is the ability to sense *infectious-self* and *non-self*, whereas for ETI it is the ability to sense microbe-mediated modifications inferred on points of vulnerability in the host. By guarding these weak points or even setting up decoys to confuse invaders, ETI is an efficient safety net for more progressed infections (van der Hoorn and Jones, 2004; Jones and Dangl, 2006).

Pathogen-associated molecular pattern perception is mediated by ligand-binding surface-exposed transmembrane pattern-recognition receptors (PRRs) of either the receptor-like kinase (RLK) or receptor-like proteins (RLPs) families. Both types of modular proteins are single-pass transmembrane proteins with extracellular domains, but where RLKs have an intracellular kinase domain; RLPs lack this cytosolic signaling domain (Monaghan and Zipfel, 2012). The archetypical bacterial PRRs are elongation factor Tu (EF-Tu) receptor (EFR), a leucine-rich repeat RLK (LRR-RLK) that recognizes the abundant cytoplasmic protein EF-Tu; and the related flagellin sensing 2 (FLS2) that recognizes flagellin, the principal component of bacterial flagella. These two PAMPs are often characterized by their minimal requirement peptide epitopes, elf18 and flg22 (Gomez-Gomez et al., 1999; Kunze et al., 2004; Boller and Felix, 2009). Sensing of the elf18 peptide appears to be restricted to the *Brassicaceae* family, while in rice recognition of EF-Tu, occurs via the EFa50 region (Furukawa et al., 2013). Interestingly, transgenic expression of *Arabidopsis* (*Arabidopsis thaliana*) EFR in *Solanaceae* species that cannot perceive EF-Tu is sufficient to confer resistance to a broad range of phytopathogenic bacteria, suggesting high conservation of the responses downstream of PAMP recognition (Lacombe et al., 2010). PTI induction leads to a series of early and late responses. The early responses occur within minutes to hours, and consist of rapid ion fluxes across the plasma membrane, an oxidative burst, activation of mitogen-activated protein kinases (MAPKs) and calcium-dependent protein kinases (CDPKs), and induction of defense-related genes. Deposition of callose, inhibition of seedling growth and PAMP induced resistance are later responses that occur within days (Boller and Felix, 2009).

## RECOGNITION OF CHITIN: AN EVOLUTIONARY ARMS RACE

**FIGURE 1:**
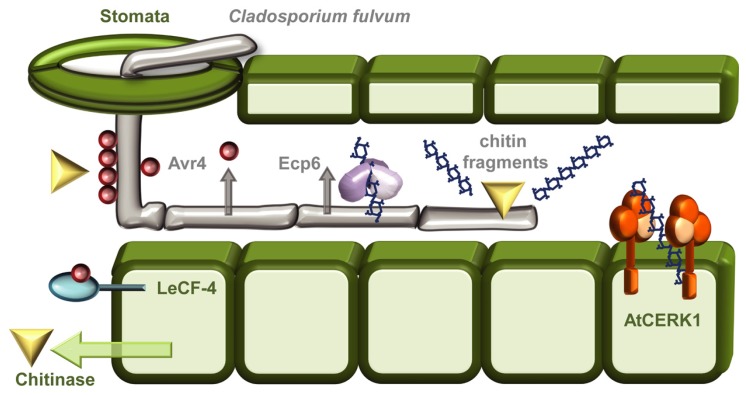
**The “arms race” between *Cladosporium fulvum* and plant hosts.** As a general protective measure against fungal infections plants respond to infection attempts by secreting chitinases into the apoplastic space. The tomato leaf mould fungus *C. fulvum* can enter its host through stomatal openings, and grows as extracellular hyphae. To shield against the action of these chitin-degrading enzymes the fungus camouflages its chitin-containing cell walls by cloaking them with the chitin-binding effector Avr4 (van den Burg et al., 2006; Hadwiger, 2013). The presence of Avr4 can be recognized by the Tomato RLP Cf-4, leading to induction of the hypersensitive response (Thomas et al., 1997; Takken et al., 1999). Chitin oligomers released by chitinases are recognized as PAMPs, in *Arabidopsis* by the RLK CERK1 (Iizasa et al., 2010; Petutschnig et al., 2010), and in rice by both OsCERK1 and the RLP OsCEBiP (Shimizu et al., 2010). To escape chitin-induced PTI *C. fulvum* can secrete Ecp6 an effector that functions as a chitin-scavenger removing the chitin oligomers released by the chitinases (de Jonge et al., 2010; Kombrink and Thomma, 2013; Sanchez-Vallet et al., 2013).

A good example of the intricate evolutionary arms race between a pathogen and its plant host concerns the polysaccharide chitin (**Figure 1**). Chitin, a homopolymer of β-(1→4)-linked *N*-acetylglucosamine (GlcNAc) units, is the major structural component of fungal cell walls, and is also a main constituent of insect exoskeletons, crustacean shells, and the eggs and gut linings of nematodes (Bueter et al., 2013; Hadwiger, 2013). Chitin is an obvious PAMP-candidate and an ideal point of attack during plant defense responses since glucosamine polymers are not found in plants. It is therefore not surprising that an evolutionarily conserved strategy toward fungi and insects in plants is based on secreting chitinases – hydrolytic enzymes, which can break down microbial chitin polymers (**Figure 1**; Hadwiger, 2013). However, as usual, countermeasures have also evolved, the biotrophic fungal pathogen *Cladosporium fulvum* combats the action of chitinases by secreting the apoplastic effector Avr4, a chitin-binding lectin that functions to protect the integrity of the fungal cell wall against chitinases (**Figure 1**; van den Burg et al., 2006). Heterologous expression of Avr4 in *Arabidopsis* or tomato camouflages the chitin thereby increasing the virulence of several fungal pathogens (van Esse et al., 2007). That is unless the host harbors Cf-4, an extracellular membrane-anchored LRR protein that mediates Avr4 perception (**Figure 1**) and activates the hypersensitive response (Thomas et al., 1997; Takken et al., 1999). In addition to this forceful degradation strategy, chitin is also recognized as a PAMP in rice (*Oryza sativa*) by a dual recognition system consisting of the lysine motif (LysM)–RLK CHITIN ELICITOR RECEPTOR KINASE-1 OsCERK1 and the LysM-RLP CHITIN OLIGOSACCHARIDE ELICITOR-BINDING PROTEIN OsCEBiP (Shimizu et al., 2010). In *Arabidopsis* there is no contribution to signaling from the major chitin-oligosaccharide binding CEBiP, and AtCERK1 seems to act alone as the chitin PRR (**Figure 1**; Iizasa et al., 2010; Petutschnig et al., 2010; Shinya et al., 2012). The biological activity of chitin oligomers depends on their size, as the highest PAMP-activity is found for heptamers and octamers. Higher oligomeric chitin fragments like octamers can bind two or more AtCERK1 and this ligand-induced dimerization activates the receptor (Liu et al., 2012). To avoid chitin-induced PTI, *C. fulvum* also secretes the evolutionally conserved LysM-containing effector extracellular protein 6 (Ecp6) during infection. Ecp6 is a scavenger of chitin fragments released by chitinases and out-competes chitin-PRR binding to avoid fungal detection (**Figure 1**; Bolton et al., 2008; de Jonge et al., 2010; Sanchez-Vallet et al., 2013). Whether Ecp6 is recognized in plants is still unknown. In all cases, this example demonstrates the continuous battle between pathogen and host and how one party’s armor is continuously being evolutionarily countered by the opponent.

## PTI SIGNALING

A vital feature of plant innate immunity is the early recognition of potential pathogens via perception of PAMPs and DAMPs. This recognition is mediated by designated surface-localized PRRs and sets in train a range of early and late responses that ensure a specialized and appropriate response is transmitted by a shared downstream pathway. Some of these steps are already mapped whereas others are yet to be discovered.

**FIGURE 2:**
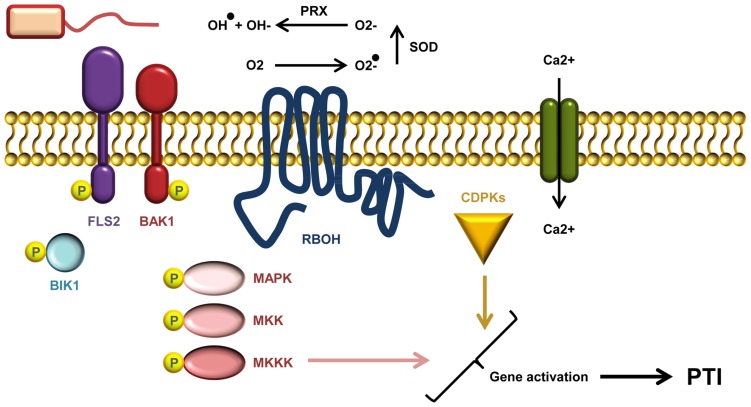
**A current model of AtFLS2 signaling.** Upon flg22 binding, a complex between FLS2, BIK1, BAK1 (and other SERKs) is formed. Complex formation triggers multiple rapid phosphorylation events resulting in BIK1 release. The signal transduction downstream of ligand perception includes a Ca^2^^+^ burst, activation CDPKs and AtRbohD required for the ROS burst, and induction MAPK cascades. Activation of CDPKs and MAPKs is required for full induction of defense genes.

After ligand recognition, several PRRs rapidly form complexes with the regulatory RLK BRI1-ASSOCIATED KINASE1 (BAK1; **Figure 2**; Monaghan and Zipfel, 2012). Indeed, complex formation between the co-receptor and the flagellin receptor FLS2 occurs within seconds of flg22 treatment (Schulze et al., 2010). BAK1 was believed to function solely as a signal enhancer rather than being involved in ligand-binding. However, it has recently been demonstrated that BAK1 acts as a co-receptor in FLS2 mediated flg22 recognition by interacting with the bound ligand (Sun et al., 2013). BAK1 belongs to the somatic embryogenesis-related kinases; a small family of five LRR-RLKs (Heese et al., 2007; Roux et al., 2011). In addition to FLS2, BAK1 has also been shown to interact with EFR and the DAMP receptors PEPR1 and PEPR2 (Krol et al., 2010; Yamaguchi et al., 2010). Apart from its association with PRRs, BAK1 is also involved in the perception of brassinosteroids through its interaction with the RLK BRASSINOSTEROID INSENSITIVE 1 (BRI1; Li et al., 2002; Nam and Li, 2002). PAMP-binding and complex formation leads to auto- and trans-phosphorylation between PRR and co-receptor, as well as the plasma membrane localized receptor-like cytoplasmic kinase BOTRYTIS-INDUCED KINASE 1 (BIK1) and related PBS1-LIKE (PBL) kinases (Lu et al., 2010; Zhang et al., 2010). BIK1 plays a central role in conveying signals from several PRRs including EFR, FLS2, CERK1, and PEPR1/PEPR2. PAMP/DAMP induced phosphorylation of BIK1 prompts dissociation from the PRR complex which may permit BIK1 to move toward and activate downstream signaling targets (Lu et al., 2010; Zhang et al., 2010; Liu et al., 2013).

## CONVEYING THE SIGNAL INTO TRANSCRIPTIONAL REPROGRAMMING

Calcium ions serve as important second messengers in eukaryotes. Both abiotic and biotic stress responses lead to rapid transient fluctuations in the concentration of intracellular calcium ions (**Figure 2**). The low basal cytosolic concentration allows rapid spatial and temporal changes in the calcium flux. Such calcium signatures are sensed by stimulus-specific Ca^2^^+^-binding sensors that can mediate the downstream signal transduction. Deciphering the calcium signatures enables the affected cells to respond appropriately to diverse stimuli (DeFalco et al., 2010). During PTI, CDPKs function as Ca^2^^+^-sensors that are required to obtain complete transcriptional reprogramming (**Figure 2**). The four functionally redundant *Arabidopsis* CDPKs, CPK4, CPK5, CPK6, and CPK11 define a sub-clade required for flg22 induced PTI responses (Boudsocq et al., 2010). The transcriptional changes effected by the CDPKs are mostly independent of the MAPK pathway (see below), but the combination of CDPKs and MAPKs is needed to activate at least four early transcriptional regulatory programs induced by flg22 (Boudsocq et al., 2010; Wurzinger et al., 2011).

An oxidative burst of reactive oxygen species (ROS) such as ·O_2_^-^, ·OH, and H_2_O_2_ is one of the early measurable events in PTI (**Figure 2**). In *Arabidopsis*, the ROS burst is facilitated by the RESPIRATORY BURST OXIDASE HOMOLOGUE D (RbohD), an NADPH oxidase (Torres et al., 2002). The activity of RbohD is dependent on calcium and phosphorylation (Ogasawara et al., 2008; Kimura et al., 2012), and interestingly RbohD is phosphorylated by CDPK5 (Dubiella et al., 2013). In potato (*Solanum tuberosum*) StRbohB is phosphorylated by the plasma membrane-localized potato StCDPK5, and expression of constitutively active StCDPK5 in *Nicotiana benthamiana* leads to induction of ROS (Kobayashi et al., 2007).

Like the CDPKs, MAPK cascades also function to transmit signals within the cell via differential phosphorylation. In such cascades MAP kinase-kinase-kinases (MAPKKKs) phosphorylate MAP kinase-kinases (MAPKKs), which in turn phosphorylate MAP-kinases (**Figure 2**; Rasmussen et al., 2012). The *Arabidopsis* MAP kinases MPK3, MPK4, MPK6 as well as the recently documented MPK11 are all activated by PAMP treatment (Rasmussen et al., 2012). They designate at least two different pathways: one that triggers MPK4 activation by a module consisting of the two MAPKKs MKK1 and MKK2, and MEKK1; and another that leads to MPK3/MPK6 activation, via MKK4 and MKK5, and possibly MEKK1 (Rasmussen et al., 2012). Activation of MAPK cascades is, together with CDPK activation, vital for obtaining the transcriptional reprogramming needed to mount a full PTI response (**Figure 2**; Boudsocq et al., 2010). Even though some genes require both MAPK and CDPK activation to be induced there seems to be a branching of PTI signals on some level (Boudsocq et al., 2010; Xu et al., 2014). Loss of the flg22-induced ROS in *Arabidopsis rbohD* does not affect MPK3 and MPK6 activation, and activation of these MAP kinases is also not required for ROS burst (Xu et al., 2014).

## FATE OF ACTIVATED RECEPTORS

Ubiquitination is a key signal for endosomal sorting of membrane proteins (Raiborg and Stenmark, 2009). Upon ligand perception FLS2 is being ubiquitinated by two PLANT U-BOX (PUB) E3 ligases PUB12 and PUB13 (Lu et al., 2011). This may well be part of a strategy to terminate the signal initiated by PAMP recognition. FLS2 is endocytosed and targeted for endocytic degradation approximately 30–60 min post-elicitation, and the activated FLS2 travels through early, late and multi-vesicular endosomes toward its vacuolar destination (Robatzek et al., 2006; Chinchilla et al., 2007; Beck et al., 2012b; Smith et al., 2014). The removal of ligand-bound receptors de-sensitizes the system after initial PAMP-treatment. After two hours the de-sensitized cells begin to be re-sensitized through *de novo* synthesis mediated replenishment of the receptor, preparing them for a new round of ligand perception (Smith et al., 2014). FLS2 has recently been shown to interact with two subunits of ENDOSOMAL SORTING COMPLEXES REQUIRED FOR TRANSPORT-I (ESCRT-I; Spallek et al., 2013). ESCRT complexes are responsible for identifying ubiquitinated vesicular cargoes, sorting them for degradation, and depositing them in intraluminal vesicles to prevent their recycling (Raiborg and Stenmark, 2009). Loss of the E3 ligases or ESCRT-I components affects flg22-triggered immune reactions underlining the importance of correct endosomal trafficking of FLS2 in sustaining a sufficient level of immunity (Lu et al., 2011; Spallek et al., 2013). In addition removal of activated receptors, trafficking might also play an integrated part in signaling transduction as part of the signaling responses may happen from endosomal compartments (Geldner and Robatzek, 2008; Beck et al., 2012a).

## LATE PTI RESPONSES

The final consequence of PTI is the induction of resistance responses that will prevent microbial colonization. Other late responses are the inhibition of seedling growth inhibition occurring within days of continuous PAMP treatment, and deposition of callose about 16 h after PAMP treatment (Boller and Felix, 2009). Callose is found during attempted fungal infections in cell wall fortifications termed papillae, structures that are assembled at fungal penetration sites (Underwood, 2012). The linear β-(1→3)-D-Glc*p* homo-polymer is produced by callose synthases; among these is the *Arabidopsis* POWDERY MILDEW RESISTANT 4 (PMR4), the predominant synthase responsible for defense-induced callose deposition (Vogel and Somerville, 2000; Nishimura et al., 2003; Ham et al., 2007; Chen and Kim, 2009). All the callose deposited in response to flg22, and most of the chitosan-induced depositions are synthesized by PMR4 (Luna et al., 2011). In addition to differences in their dependency on PMR4, callose depositions induced by these two PAMPs also differ in their requirements for other signaling components as only the flg22-induced callose depends on RbohD (Luna et al., 2011). Callose depositions like the papillae are an important feature of immunity, and are thought to reinforce the cell wall at fungal penetration sites to impede infections (Underwood, 2012). In agreement with this, overexpression of PMR4 in *Arabidopsis* results in enlarged callose deposits, and gain of penetration resistance against both virulent and non-adapted powdery mildew strains (Ellinger et al., 2013; Naumann et al., 2013). Absence of callose depositions also leads to susceptibility toward the non-adapted hemibiotrophic bacteria *Pseudomonas syringae* pv. *phaseolicola* (Ham et al., 2007). Surprisingly the *pmr4* mutant was identified by its increased powdery mildew resistance, a phenotype that is dependent on the defense phytohormone salicylic acid (Vogel and Somerville, 2000; Nishimura et al., 2003; Huibers et al., 2013). These observations are counterintuitive both with respect to the pattern of callose deposition during defense induction, and the recent evidence from overexpression lines, supporting a role for callose in the plants defensive strategy (Vogel and Somerville, 2000; Ellinger et al., 2013; Naumann et al., 2013). One explanation for such a discrepancy could be that PMR4 is guarded by a resistance gene. Accordingly, loss of PMR4 might be perceived as a breach on the plants defensive fences and thus set about induction of ETI via elevation of salicylic acid.

## THE ROLE OF PLANT CELL WALLS IN MICROBIAL INTERACTIONS

**FIGURE 3:**
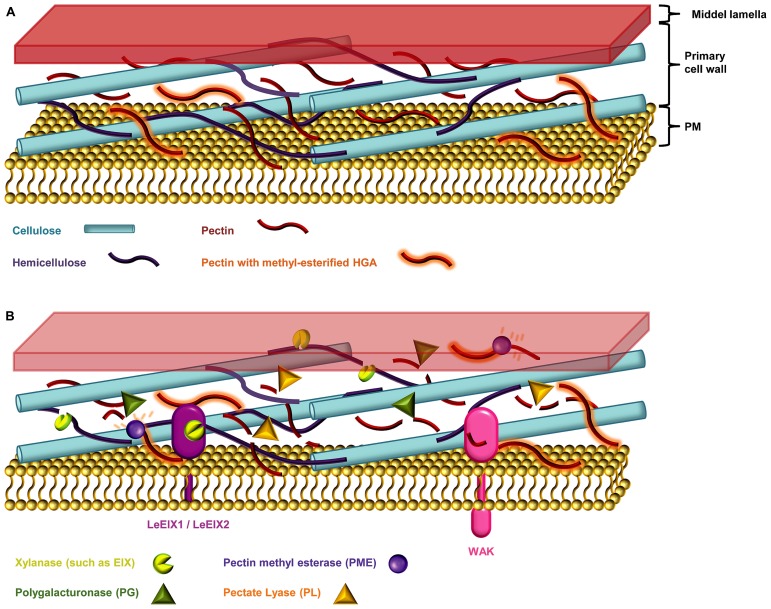
**The plant cell wall. (A)** The primary cell wall is constructed of a web of cellulose micro-fibrils, hemicellulose polysaccharides, and the hetero-polysaccharide pectin (Endler and Persson, 2011). **(B)** Necotrophic pathogens devour their hosts by degrading the plant cell wall, and secreting necrosis-inducing proteins and toxins (Davidsson et al., 2013). The host entry step of such microbes is thought to happen mainly via secretion of cell wall degrading enzymes such as cellulases, pectinases, and hemicellulases (Choquer et al., 2007; Davidsson et al., 2013). Xylanase, degrades the linear backbone of the predominant hemicellulose xylan into xylose residues (Belien et al., 2006; Scheller and Ulvskov, 2010). Pectinases such as polygalacturonases (PGs) and pectate lyases (PLs) degrades the pectic homogalacturonan (HGA) backbone. The HGA in newly synthesized pectin is methyl-esterified, which protects it from degradation by pectinases. De-esterification of HGA by the action of microbial pectin methyl esterases (PMEs) enables access PGs and PLs (Annis and Goodwin, 1997; Wolf et al., 2009; Lionetti et al., 2012). Endo-PGs cleave un-esterified regions while concomitantly releasing the oligogalacturonides (OGAs), oligomeric fragments of the HGA backbone (Annis and Goodwin, 1997; Ferrari et al., 2013). The wall-associated kinases (WAKs) are DAMP sensors that monitor the integrity of pectin by sensing the presence of OGAs (Ferrari et al., 2013). Plants can counter the cocktail of cell wall degrading enzymes by producing proteinaceous inhibitors such as the xylanase inhibitors and PG inhibitor proteins (PGIPs; Belien et al., 2006).

Plant cell walls not only provide structure to the plant body but also act as barriers against biotic and abiotic stresses. The cell wall, sometimes covered with a cuticle, is usually the first obstacle encountered by pathogens, and to penetrate this barrier microbes have evolved an arsenal of wall degrading enzymes which are key virulence factors (**Figure 3**; Nuhse, 2012; Davidsson et al., 2013).

Most plant cell walls are based on a co-extensive load-bearing network of cellulose micro-fibrils cross-linked with hemicelluloses (**Figure 3**; Fry, 2004; Scheller and Ulvskov, 2010; Pauly et al., 2013). In the primary walls of the growing parts of plants, this network is embedded in a matrix of pectic polysaccharides. In the secondary cell walls of mature, non-growing tissues pectin is less abundant but walls are reinforced with lignin (Endler and Persson, 2011). Although most cell walls are based on these main components they differ considerably in their fine structures and three dimensional architectures. This heterogeneity is reflected in the diversity of strategies that pathogens have evolved to breach them, including the secretion of numerous glycosyl hydrolases (Annis and Goodwin, 1997). In response to an attack, plants may deposit certain reinforcing polymers, notably callose, phenolic complexes, and employ toxic compounds (Huckelhoven, 2007). However, these physical and chemical responses are only one part of the cell walls role in defense and the extensive ability of pathogens to degrade cell wall component has a cost. Disturbance of cell wall integrity (which may also cause deformation of adjacent portion of the plasma membrane) and the release of degradation fragments are monitored by plants and changed cell wall status is an important trigger for defense mechanisms (Hematy et al., 2009). Despite the undoubted importance of cell walls in plant defense there are many aspects that are poorly understood. For example, most work has focused on a limited set of cell wall polymers (notably callose, extensins, and lignin) and the potential roles of many other cell wall components are obscure. Another largely unexplored aspect of cell walls and defense is the evolution of and co-evolution of defense strategies (Sørensen et al., 2010). Discussed below are the major cell wall components in the context of pathogenic responses to them, and the prospects for advancing our understanding using emerging microarray technologies.

## CELLULOSE

Tightly packed crystalline cellulose micro-fibrils serve a primary structural function in the cell wall (Endler and Persson, 2011; Nuhse, 2012). The fibrils are composed of hydrogen-bonded β-(1→4)-D-Glc*p* chains, and have a condensed nature that makes them generally resistant to degradation via glycosyl hydrolases. However, recent work has highlighted the role of another class of oxidative enzymes, the lytic polysaccharide monoxygenases that have the capacity to degrade recalcitrant crystalline cell wall components, including cellulose (Vaaje-Kolstad et al., 2010; Hemsworth et al., 2014). Cellulose micro-fibrils are synthesized by large multimeric complexes containing cellulose synthase catalytic subunits (CESAs; Endler and Persson, 2011). It is generally considered that in *Arabidopsis*, and probably other angiosperms, there are two CESA subfamilies with distinct roles. Whilst CESAs 1, 3, and 6 are responsible for the formation of cellulose in primary walls, CESAs 4, 7, and 8 synthesize cellulose in secondary walls (Endler and Persson, 2011). However, recent work has demonstrated that some functional cross over exists across these groups *in vitro* although it is not clear if these new CESA pairings are significant *in planta* (Carroll et al., 2012).

As in many cases where cell wall integrity is perturbed, compensatory mechanisms have evolved to limit the effects of disturbance to cellulose structure or deposition. Cellulose deficient mutants typically exhibit increased lignification (Cano-Delgado et al., 2003; Hamann, 2012). Interestingly, these changes appear to have effects beyond the purely structural. Such mutants also display enhanced defense responses (Hamann, 2012) as mutants in the primary wall *CESA3* are more resistant toward powdery mildew (Ellis and Turner, 2001; Cano-Delgado et al., 2003). Additionally defects in the secondary wall CESAs; CESA4, CESA7, and CESA8, also lead to elevated resistance against broad-host necrotrophic pathogens like the fungus *Plectosphaerella cucumerina* and the soil-borne bacterium *Ralstonia solanacearum* (Hernandez-Blanco et al., 2007). Consistent with the genetic evidence, similar results have also been described for treatment with the cellulose biosynthesis inhibitor isoxaben (Hamann et al., 2009; Hamann, 2012). In *Arabidopsis*, isoxaben-mediated wall damage and lignin production is induced via an RbohD-dependent mechanism, and fine-tuned through a jasmonic-acid-dependent negative feedback loop (Hamann et al., 2009; Denness et al., 2011). The defense associated cellulose deprivation phenotypes suggest that the cell wall damage triggers defense responses, and suggest the presence of a tight cell wall integrity monitoring system. The RLK THESEUS1 (THE1) seems to serve a role in this as it has been implicated in the response to cell wall damage. The *the1* mutant was identified as a suppressor of the *cesA6* null allele *procuste1-1.* Mutations in *THE1* reduce the ectopic lignification of several cellulose deficient mutants (Hematy et al., 2007).

## HEMICELLULOSES

Hemicelluloses are a diverse group of polysaccharides usually characterized by having a β-(1→4)-linked backbone of mannose, glucose, or xylose. The central role of hemicelluloses is to fortify the cell wall by interaction with cellulose and sometimes lignin (Scheller and Ulvskov, 2010; Endler and Persson, 2011). Xylans are the predominant hemicelluloses in secondary plant cell walls. The common characteristic of xylans is a backbone of repeating β-(1→4)-D-Xyl*p* residues most often substituted by Ara*f* and Glc*p*A (Scheller and Ulvskov, 2010). The arabinosyl residues can in addition contain ferulic acid groups esterified to the O-5 position of the carboxyl group (Smith and Hartley, 1983). These ferulate esters can be oxidatively cross-linked to lignin possibly incorporating xylan into the lignin complex to advance strengthening of the network (Tanner and Morrison, 1983; Mcneil et al., 1984; Harris and Stone, 2009). Some phytopathogenic microbes secrete xylanases, which are enzymes that can degrade the linear xylan backbone into xylose units (Belien et al., 2006). Hemicellulose breakdown by xylanases weakens the cell wall and enables the microbe to breach it (Belien et al., 2006). Fungi of the *Trichoderma* spp. produce the ETHYLENE-INDUCING XYLANASE (EIX) that is recognized as a PAMP (Furman-Matarasso et al., 1999; Belien et al., 2006). In tomato (*Lycopersicum esculentum*), EIX is sensed by the cell wall-derived RLPs LeEix1 and LeEix2 (**Figure 3**; Ron and Avni, 2004).

In addition to feruloylation cell wall glycans can also be subjected to methylation and acetylation. REDUCED WALL ACETYLATION 2 (RWA2) has been shown to be responsible for the acetylation of numerous pectic and non-pectic polymers in *Arabidopsis*, and *rwa2* knock-out mutants have (Mcneil et al., 1984) increased resistance toward *Botrytis cinerea* (Manabe et al., 2011). Furthermore de-acetylation of xyloglucan and pectin in transgenic plants exhibit increased accessibility to degrading enzymes which could be part of a defense strategy to release oligosaccharides which can act as defense elicitors (Pogorelko et al., 2013).

## PECTIN

The complex hetero-polysaccharide pectin is a major cell wall matrix component that is abundant in primary cell walls. It is composed of a series of structurally distinct domains acting as backbones or side chains to the pectic polysaccharides (Wolf et al., 2009). Back bones are comprised either of contiguous α-(1→4)-D-Gal*p*A residues (homogalacturonan or HGA) or repeating dimers of α-(1→4)-D-Gal*p*A-α-(1→2)-L-Rha*p* residues (rhamogalacturonan). Within the pectin complex especially HGA appears to have special significance in the context of defense responses. HGA can be methyl esterified at C6 and acetylated at C2–C3 and regulation of these substitutions enable plants to fine-tune the functionality of HGA to suit prevailing local requirements. For example non-esterified HGA carrying negatively charged free carboxyl groups may be subject to cross-linking via calcium leading to the formation of gels that are required for cell adhesion and other structural roles (Cabrera et al., 2008).

Some of the first enzymes that phytopathogenic fungi secrete during infections are endo-polygalacturonases (PGs) which cleave HGA (**Figure 3**), thereby degrading cell wall integrity and aiding pathogen access (Annis and Goodwin, 1997). Moreover, HGA degradation releases oligogalacturonide (OGA) fragments from the backbone, which act as potent defense response elicitors (Galletti et al., 2009). The WALL-ASSOCIATED KINASES (WAKs) are thought to be DAMP sensors that monitor the integrity of pectin by detecting the presence of OGAs with a degree of polymerization between 10 and 15 (Ferrari et al., 2013). In the context of degradation by microbial enzymes the level of HGA methyl esterification is critically important. Endo-PGs and pectate lyases (PLs) preferentially cleave non-esterified HGA and these enzymes frequently act in concert with pectin methyl esterases (PMEs) that de-methyl esterify HGA to create endo-PG and PL cleavage sites (**Figure 3**). Moreover, another class of microbial enzymes – the pectin lyases act preferentially on highly methyl esterified HGA.

Homogalacturonan and the microbial enzymes that degrade it are good examples of how the high complexity of cell wall components is countered by sets of highly specific microbial enzymes. Nevertheless, the role of pectin in plant defense cannot only be viewed in straightforward terms of polysaccharide integrity versus enzymatic breakdown. An example is provided by the elevated activity of certain endogenous *Arabidopsis* PMEs in response to infection with the necrotrophic fungus *Alternaria brassicicola*, the hemibiotrophic bacterial pathogen *P. syringae pv. maculicola* ES4326, or by treatment with PAMPs (Bethke et al., 2014). The increase in PME activity in such infected plants leads to a concomitant decrease in methyl esterification of HGA, which doubtlessly renders the HGA more susceptible toward endo-PGs (Bethke et al., 2014). It seems counterintuitive to increase the activity of an endogenous wall degrading enzyme during a pathogen attack as this could aid microbial entry. One explanation is that the enhanced production of OGAs – and thereby an associated potential activation of DAMP signaling – is worth the price of a decrease in HGA integrity. It is also important to consider the broader picture of the 66 PMEs in *Arabidopsis* in terms of redundancy and spatiotemporal regulation. For example, in the case of *A. brassicicola* it is worth noting that the observed increase in PME activity occurred rather late after pathogen challenge. With this in mind it may be the case that the purpose of enhanced PME activity is localized HGA de-methyl esterification and the concomitant formation of gel structures to bolster damaged walls (Bethke et al., 2014).

Another noteworthy insight into the roles of pectin in defense is provided by the loss of function mutants of the endogenous plant enzyme POWDERY MILDEW RESISTANT 6 MUTANT (PMR6; Vogel et al., 2002). PMR6 has similarity to PLs, and loss of function leads to enhanced levels of intact pectin and but also lower levels of potential OGA release. One explanation for the enhanced resistance to powdery mildew in *pmr6* is that increased levels of inaccessible HGA *per se* provide more protection. But, the fact that *pmr6* only exhibits this elevated resistance against certain powdery mildew species (*Erysiphe cichoracearum* and *E. orontii*) implies a less generic and more subtle effect. For example, it is possible that alterations in *pmr6* cell wall composition make these mutants poor hosts specifically for *Erysiphe* spp. It certainly seems highly unlikely that a plant gene would have evolved to serve the needs of a pathogen, and the pleotropic effects observed in *pmr6* are consistent with a role as a susceptibility factor. It is plausible that *pmr6* resistance is a specialized form of disease resistance, possibly based on the loss of a host susceptibility gene required by the pathogen for growth and development (Vogel et al., 2002).

## LIGNIN

Lignin is a phenolic polymer mainly deposited in secondary cell wall during the last stages of cell differentiation. It displaces the aqueous phase of the cell wall, encasing cellulose and matrix polysaccharides and providing enhanced mechanical strength and a water-impermeable barrier (Albersheim et al., 2011). Lignin is also required for reinforcing vascular cells that transport water under negative pressure as a result of transpiration. The importance of lignin in these tissues is demonstrated by vascular collapse in lignin deficient plants (Piquemal et al., 1998; Jones et al., 2001). Lignin is built from monolignols, with up to three different types in higher plants which appear to be incorporated into the lignin polymer in a non-predictable fashion (Martone et al., 2009; Vanholme et al., 2010). This apparently random pattern of synthesis may be significant in relation to microbial enzymes which have typically evolved to break polymers with structurally consistent cleavage sites (Sarkar et al., 2009).

Lignin has multiple roles in plant defense and lignin, or lignin-like phenolic polymers are often rapidly deposited in response to both biotic and abiotic stresses (Sattler and Funnell-Harris, 2013). Lignin not only acts as physical barrier to pathogen invasion, but the phenylpropanoid pathway responsible for lignin biosynthesis may also be recruited for defense purposes. For example, this pathway underpins the synthesis of other phenolic compounds including phytoalexins, stilbenes, coumarins, and flavonoids – some of which have been implicated in plant defense (Weiergang et al., 1996; Dicko et al., 2005; Lozovaya et al., 2007). Indeed, there is evidence that in some plants, salicylic acid which is known to be a key component of some defense pathways, may also be produced by the phenylpropanoid pathway (Ruuhola et al., 2003; Pan et al., 2006). Understanding the role of lignin in plant defense has received considerable attention in the context of engineering plants for use as bioenergy feedstocks. Lignin contributes substantially to the recalcitrance of cell walls to deconstruction into fermentable sugars and for this reason reducing lignin content has become an important goal for feedstock biotechnology research. However, since lignin is also required for plant defense there is probably a lower limit for lignin reduction beyond which plant would become unacceptably vulnerable to pathogens (Sattler and Funnell-Harris, 2013). The roles of lignin in defense and the importance of this for bioenergy feedstocks are reviewed in (Sattler and Funnell-Harris, 2013).

## INHIBITORS OF MICROBIAL WALL DEGRADING ENZYMES

Often, an infection involves a multifaceted strategy. In the case of *B. cinerea* the host entry step is thought to happen mainly via secretion of cell wall degrading enzymes such as a PMEs, endo-PGs, and endo-β-1,4-xylanase often encoded by multi-genic families (Choquer et al., 2007). Cellulases, pectinases, and hemicellulases are also secreted from bacteria like the *Pectobacterium* and *Dickeya* genera necrotrophic soft rot enterobacteria (Davidsson et al., 2013). At first glance, plants would seem defenseless against such savage enzyme cocktails, however, they can counter by producing proteinaceous inhibitors of cell wall degrading enzymes such as the xylanase inhibitors and PG inhibitor proteins (PGIPs; Belien et al., 2006). The cunning strategy of making PGIPs is not to inhibit pectin degradation entirely, but to shift it toward producing longer OGAs that can be sensed as DAMPs (Federici et al., 2006).

## THE CHALLENGES OF MONITORING CELL WALL RESPONSES TO PATHOGENS

Even from a concise overview such as provided above, it is clear that analyzing the subtle, complex and often rapid changes that affect cell walls in response to pathogens is a formidable technical challenge. In contrast to proteins and nucleotides, the polysaccharides from which cell walls are primarily made cannot readily be sequenced, synthesized, or expressed. Although cell walls are usually physically tough, they are also typically highly plastic and can undergo rapid modifications in response to specific local conditions, even within cell wall micro-domains. The analytical difficulties are compounded by the sheer complexity and multilayered nature of host/pathogen interactions, and the differing nature of these interactions depending on developmental stage, organ, or tissue. Conventional biochemical techniques for carbohydrate analysis, for example methylation analysis and ion exchange chromatography, are powerful but low throughput and usually require large amounts of material. They also involve the complete or partial destruction of cell walls into their component parts – which inevitably means that information about three dimensional cell wall architectures is lost. Molecular probes, for example monoclonal antibodies (mAbs) and carbohydrate binding modules (CBMs) have been invaluable for advancing our understanding of plant cell walls *per se*, and have immense potential for providing insight into host pathogen interactions at the cellular and subcellular levels. There is an extensive repertoire of probes directed against plant cell wall components (Lee et al., 2011), but this is not matched by probes for pathogens and their associated effectors. For example, our understanding of the precise role of chitosan (the de-acetylated form of chitin) during pathogenesis is hampered by a lack of suitable probes (Sorlier et al., 2003; Hoell et al., 2010; Baker et al., 2011).

One technique that appears to offer considerable promise for plant/pathogen interaction research is based on carbohydrate microarrays (Moller et al., 2007). This technique combines the specificity of mAbs and CBMs with the multiplexed analysis capacity of robotically produced microarrays, and simultaneously provides information about the relative abundance of multiple epitopes present in large sample sets (Tyler et al., 2014). This approach has been extensively used for cell wall analysis in numerous contexts (Tronchet et al., 2010; Sørensen et al., 2011; Fangel et al., 2012; Moore et al., 2014; Zhang et al., 2014), but has only been used in a very limited way for plant defense research (Nguema-Ona et al., 2013). Typically, at least 20 mAbs or CBMs are used in a single analysis, and several 100 samples can be processed in 2 days. Analysis starts with the preparation of cell wall material (although native plant material can also be used) which is then treated with a series of solvents to release cell wall components. For example, extraction with 1,2-diaminocyclohexanetetraacetic acid followed by sodium hydroxide is expected to release pectins and hemicellulose respectively. These extractions are then printed onto a suitable surface such as nitrocellulose membrane or modified glass slides using a microarray robot, and the resultant arrays probed with mAbs and/or CBMs and then quantified. The fact that relatively small amounts of cell wall material are needed (0.5–10 mg) and the ability to rapidly analyze large numbers of samples are attractive attributes in the context of plant/pathogen interaction research. Currently, the analysis of cell walls in the context of plant defense is often limited to polymers with well characterized roles, for example extensins and callose. The described technology can potentially provide the means to greatly increase the scope of cell wall analysis, both in terms of the number of cell wall components analyzed and the number of variables in an experimental set up. Our initial experiments indicate that glycan arrays are powerful tools for monitoring the progression of cell wall changes during defense responses and are well suited to the parallel analysis of, for example, multiple mutants, elicitors, or conditions. Furthermore we anticipate that analysis of cell wall glycomes will be integrated with transcriptomic analysis of defense related genes using the same material. Moreover, although the technique has mostly been applied to carbohydrate analysis, the same approach could be applied to investigate proteins and their conjugates of relevance to pathogenic interactions. So long as analytes can be extracted and immobilized, and providing that probes are available the scope of analysis can be extended.

## CONCLUDING REMARKS

Our current knowledge of the interactions between plants and pathogens at the plant cell wall level, and how this affects downstream signaling is still limited. To obtain more insight we need to expand our understanding of the full repertoire of cell wall modifications that occurs during microbial interactions. Carbohydrate microarrays could be used to monitor the complex interplay between microbes and plants, and may reveal additional, perhaps more subtle, cell wall modifications to those documented so far. This technique enables rapid and multiplexed analysis of multiple changes in wall composition and could be used as a tool to provide new insight into the dynamic nature of host/pathogen interactions.

## Conflict of Interest Statement

The authors declare that the research was conducted in the absence of any commercial or financial relationships that could be construed as a potential conflict of interest.
